# Patellar tendon properties distinguish elite from non-elite soccer players and are related to peak horizontal but not vertical power

**DOI:** 10.1007/s00421-018-3905-0

**Published:** 2018-06-02

**Authors:** Conall F. Murtagh, Michael Stubbs, Jos Vanrenterghem, Andrew O’Boyle, Ryland Morgans, Barry Drust, Robert M. Erskine

**Affiliations:** 1 School of Sport and Exercise Sciences, Liverpool John Moores University, Liverpool, L3 3AF UK; 2 Liverpool Football Club, Liverpool, UK; 3 Department of Rehabilitation Sciences, KU Leuven, University of Leuven, 3000 Leuven, Belgium; 4 Football Association of Wales, Cardiff, Wales UK; 5 Institute of Sport, Exercise and Health, University College London, London, UK

**Keywords:** Patellar tendon, Elongation, Strain, Stiffness, Young’s modulus, Unilateral, Countermovement jump

## Abstract

**Purpose:**

To investigate potential differences in patellar tendon properties between elite and non-elite soccer players, and to establish whether tendon properties were related to power assessed during unilateral jumps performed in different directions.

**Methods:**

Elite (*n* = 16; age 18.1 ± 1.0 years) and non-elite (*n* = 13; age 22.3 ± 2.7 years) soccer players performed vertical, horizontal-forward and medial unilateral countermovement jumps (CMJs) on a force plate. Patellar tendon (PT) cross-sectional area, elongation, strain, stiffness, and Young’s modulus (measured at the highest common force interval) were assessed with ultrasonography and isokinetic dynamometry.

**Results:**

Elite demonstrated greater PT elongation (6.83 ± 1.87 vs. 4.92 ± 1.88 mm, *P* = 0.011) and strain (11.73 ± 3.25 vs. 8.38 ± 3.06%, *P* = 0.009) than non-elite soccer players. Projectile range and peak horizontal power during horizontal-forward CMJ correlated positively with tendon elongation (*r* = 0.657 and 0.693, *P* < 0.001) but inversely with Young’s modulus (*r* = − 0.376 and − 0.402; *P* = 0.044 and 0.031). Peak medial power during medial CMJ correlated positively with tendon elongation (*r* = 0.658, *P* < 0.001) but inversely with tendon stiffness (*r* = − 0.368, *P* = 0.050).

**Conclusions:**

Not only does a more compliant patellar tendon appear to be an indicator of elite soccer playing status but it may also facilitate unilateral horizontal-forward and medial, but not vertical CMJ performance. These findings should be considered when prescribing talent selection and development protocols related to direction-specific power in elite soccer players.

## Introduction

During the course of a match, an elite soccer player may perform up to ~ 119 maximal accelerations, ~ 35 sprints (Bradley et al. 2010), ~ 50 forceful changes of direction (Withers et al. 1982), and ~ 16 vertical jumps (Reilly and Thomas 1976). This activity profile implies that the most common type of powerful actions elicited involve the successive combination of eccentric and concentric muscle actions, also known as the stretch-shortening cycle (Cavanagh and Komi 1979). Indeed, elite soccer players have been previously shown to outperform non-elite soccer players during unilateral jumping activities in different directions, which induce stretch-shortening cycle actions in the muscle–tendon unit of the lower limb (Murtagh et al. 2017). The viscoelastic properties of the tendon affect the interaction between the contractile and elastic elements of the muscle–tendon unit, and are thought to influence performance during stretch-shortening cycle activities (Bojsen-Møller et al. 2005). However, the importance of tendon properties for determining elite soccer playing status, and their contribution to soccer-associated power capabilities, has not yet been investigated.

A comparison of tendon properties in populations of well trained and untrained, or different sporting performance levels, may provide an insight into the importance of these characteristics for high-level sports performance (Tillin et al. 2010). Within this context, high- (Kubo et al. 2011) and intermediate-level (Kubo et al. 2000b) sprinters have a more compliant vastus lateralis (VL) aponeurosis than untrained individuals. More specifically, significant correlations have been reported between 100 m race performance and VL aponeurosis compliance (Kubo et al. 2000b) and maximal elongation (Stafilidis and Arampatzis 2007). This suggests that greater elongation of the VL tendon–aponeurosis complex allows the utilisation of greater energy storage and increases the shortening velocity of the knee-extensor muscle–tendon unit during sprint running. Results from some vertical jump studies are in accordance with these findings, and report that VL aponeurosis stiffness correlated inversely with the calculated difference in jump height between bilateral vertical countermovement jumps and squat jumps. These data suggest that the greater compliance of the VL tendon–aponeurosis complex facilitates the augmentation in jump performance with a countermovement (i.e., when comparing bilateral vertical countermovement jumps *vs*. squat jumps) (Kubo et al. 1999). In contrast, Bojsen-Møller et al. (2005) demonstrated that VL aponeurosis stiffness correlated positively with bilateral vertical countermovement jump height and knee-extensor isometric rate of torque development, thus suggesting that a stiffer tendon–aponeurosis complex contributes to enhanced muscle output during high force isometric and dynamic bilateral vertical jumping tasks (Bojsen-Møller et al. 2005). In agreement, Wiesinger et al. (2016) directly assessed the properties of the patellar tendon in vivo and showed significantly greater tendon stiffness in elite ski jumping athletes compared to controls. However, Wiesinger et al. (2016) did not assess sport-specific performance directly and the previous research investigating the contribution of tendon properties to jump performance is inconclusive. Moreover, the methods utilised in the previous research investigating the association between tendon properties and sport-specific performance were limited, as they measured the properties of the VL aponeurosis rather than the patellar tendon and, therefore, had to apply a series of corrections to estimate tendon elongation (Kubo et al. 2011; Bojsen-Møller et al. 2005; Stafilidis and Arampatzis 2007). Assessing in vivo human tendon properties directly via ultrasound and isokinetic dynamometry (Seynnes et al. 2009; Hansen et al. 2006; Wiesinger et al. 2016), on the other hand, would enable a more precise investigation of a potential relationship between tendon properties and sport-specific measures of physical performance.

As the vast majority of explosive actions performed during elite soccer match-play involve a single legged push-off (Bradley et al. 2010; Withers et al. 1982), and unilateral CMJs in different directions differentiate elite from non-elite soccer players (Murtagh et al. 2017), such assessments can be used to assess soccer-associated power (Murtagh et al. 2017). Indeed, we have previously shown that multi-directional unilateral CMJs assess independent power qualities (Murtagh et al. 2017) and are underpinned by discrete neuromuscular factors (Murtagh et al. 2018). However, the importance of tendon properties in elite soccer and their contribution to unilateral CMJ performance in different directions is unknown. Such information could inform the specific detail of soccer talent identification and development protocols relative to the different properties of the PT. Therefore, the aims of this study were (1) to investigate whether PT properties differed between elite and non-elite soccer players; and (2) to establish if relationships existed between the morphological, mechanical, and material properties of the PT and unilateral jump performance in different directions in soccer players.

## Methods

### Subjects

Twenty-nine male soccer players volunteered to take part in this study, which was approved by Liverpool John Moores University Ethics Committee and complied with the Declaration of Helsinki. Participants provided written informed consent prior to being assigned to two groups according to their level of competition. The elite soccer player group (*n* = 16; age 18.3 ± 1.3 years; height 1.81 ± 0.07 m; body mass 76.2 ± 9.7 kg) consisted of players from an English Premier League football academy, who regularly participated at U18- and U21-level training and matches. The average weekly training/match content for elite soccer players was: 1 ± 1 soccer matches, 4 ± 1 soccer training sessions, 3 ± 1 gym-based resistance training sessions (two upper body and one lower body), and 4 ± 1 non-resistance training gym sessions (consisting of injury prevention, muscle activation and movement training). For more detail on the elite soccer player training frequency/type details, please see our recent study (Brownlee et al. 2018). The average weekly training content for the non-elite soccer players was: 1 ± 1 soccer matches, 2 ± 1 soccer training sessions, 1 ± 1 gym-based resistance training sessions, and no player reported participating in any non-resistance training gym sessions. The inclusion criteria for the non-elite soccer player group (*n* = 13; age 22.4 ± 1.7 years; height 1.74 ± 0.06 m; body mass 72.6 ± 6.6 kg) was to participate in at least 1 h per week of competitive soccer (11-a-side or five-a-side), and 1 h per week of soccer-specific or fitness-based training. Non-elite participants were excluded if they did not meet these inclusion criteria or had previously played soccer at academy, semi-professional, or professional level. All participants had been free of any injury to the lower body within the previous 3 months and had not previously sustained a serious knee or ankle injury that may be aggravated during testing procedures, or cause an adverse effect on performance. Participants were fully familiarised with all testing procedures in a separate session and were asked to complete a physical activity and health questionnaire prior to the study for screening purposes. This questionnaire allowed us to ascertain if each potential participant satisfied the specific inclusion and exclusion criteria.

### Experimental design

All participants attended the laboratory on two occasions, separated by between 3 and 7 days. The first session enabled the participants to be familiarised with the assessment protocol, which consisted of three unilateral CMJs in the vertical, horizontal-forward, and medial directions on each leg, and two knee-extensor ramp maximum voluntary contractions (RMVCs) on an isokinetic dynamometer. All CMJs were visually demonstrated to the participants by the investigator. This session was also used to determine the superior jumping leg (defined as the limb that produced the highest ground reaction force during a unilateral vertical CMJ). During the second session, the participants performed all assessments for CMJ and tendon properties on the dominant leg, and the results from these assessments were used for subsequent analysis. To minimise the influence of previous activity, the testing was performed at least 48 h following any high-intensity exercise. Participants were also instructed not to consume alcohol in the 48 h before testing sessions, and to consume no more than 3 mg/kg caffeine (250 mg as an absolute caffeine guideline, e.g., no more than one cup of coffee) in the 5 h prior to the beginning of testing sessions.

### Methodology

#### Countermovement jumps

On arrival at the laboratory for the second session, all participants had their height and body mass measured. Participants performed three trials of each CMJ type (with 60 s recovery between trials within a single CMJ type, and 180 s between jump types), thus performing a total of 18 CMJs (9 unilateral jumps on each leg). The methods for the performance and data analysis of unilateral CMJs have been explained in detail previously (Murtagh et al. 2017). As recommended by Jaric et al. (2005), peak power outputs achieved during CMJs were isometrically scaled to body mass (peak power divided by body mass^0.67^). The key performance variables for the unilateral vertical CMJ were jump height [calculated from the impulse–momentum relationship derived take off velocity and equation of constant acceleration methods (Dowling and Vamos 1993)] and peak vertical power isometrically scaled to body mass^0.67^ (peak V-power). The key performance variables for horizontal-forward and medial CMJs were projectile range [calculated using equations of constant acceleration (Grimshaw et al. 2004)], peak V-power, and peak horizontal power isometrically scaled to body mass [(peak H-power) for horizontal-forward CMJs only] or peak medial power isometrically scaled to body mass [(peak M-power) for medial CMJs only]. Projectile range was used as the criterion performance measure for horizontal-forward and medial CMJs as, unlike when measuring jump distance using a measuring tape, projectile range is not affected by airborne and landing technique and better represents the propulsive phase of the jump (Meylan et al. 2012).

#### Tendon morphology

Patellar tendon length, cross-sectional area (CSA), and elongation measurements were performed using ultrasonography (MyLab30, Esaote, Genoa, Italy) with the knee joint set at 90° knee flexion (full extension = 0°) and the hip joint set at 85° (supine = 180°). The PT resting length, defined as the distance between the patella apex and the point at which the tendon inserts into the tibial tuberosity, was determined by positioning the 40-mm-wide, 10–15 MHz linear transducer in the sagittal plane over the PT and marking the location of the patella apex (0% tendon length) and tibial tuberosity (100% tendon length) on the skin with a permanent marker pen. Three more locations were then marked on the skin over the tendon (25, 50, and 75% tendon length) to enable PT CSA to be measured from these axial images using Image Analysis Software (ImageJ V.1.45s, National Institute of Health, MD, USA). Transverse scans were taken at each segment until an acceptable scan, in which the borders of the whole PT cross section could be clearly identified. This was generally achieved between one and three attempts. As the stress imposed upon the PT during soccer activity is influenced by the forces exerted by the quadriceps femoris muscle, in the absence of a published isometric power law for this variable, PT CSA measurements were also isometrically scaled to body mass (tendon cross-sectional area divided by body mass^0.67^; sCSA) (Seynnes et al. 2011).

#### Tendon elongation during RMVC

Details and reliability of the measurements have been documented previously (Seynnes et al. 2009; Hansen et al. 2006). In short, the mechanical properties of the tendon were assessed by measuring the PT elongation during RMVCs. Prior to performing two RMVCs, a 2-mm-wide strip of surgical tape (3M, Neuss, Germany) acted as an echo-absorbent marker and was placed on the skin transversely over the tendon as a reference point at ~ 30% tendon length from the proximal end. Immediately before the RMVCs, participants performed a series of five submaximal isometric contractions to ensure preconditioning of the tendon. The linear transducer was then placed in the sagittal plane over the PT (~ 1 cm of the probe above the patellar apex and ~ 3 cm below, incorporating the echo-absorbent marker). Similar to the previous research that prescribed a specific time period for ramped isometric contractions when measuring tendon properties (Hansen et al. 2006; Seynnes et al. 2009; Malliaras et al. 2013), the RMVCs lasted 6 s in total with 2 min rest between each contraction. Loading rates (Nm/s) were, therefore, dependent on the participant’s maximal voluntary force capacity. Visual feedback of force production was displayed on a screen in front of the participants to ensure that all RMVCs were performed at a constant loading rate. Trials were discarded when the torque trace deviated too much from the required linear pattern upon visual inspection (Helland et al. 2013). Torque data during the RMVC were sampled using data acquisition software (AcqKnowledge, Biopac Systems Inc., Goleta, CA, USA). Ultrasound video sequences were recorded at 25 Hz during the RMVC and were synchronised with the RMVC torque data via the administration of a square wave pulse, which was visible simultaneously on the AcqKnowledge software and the ultrasound monitor (ECG signal). It is acknowledged that this technique may lead to an underestimation of the PT elongation due to unmonitored tibial movements (Onambélé et al. 2007). Unfortunately, the 4-cm-wide ultrasound transducer did not enable simultaneous scanning of both proximal and distal insertions of the PT. However, because the major portion of the tendon was scanned in the same way in all participants, this technical compromise does not invalidate the comparative outcome of the data. Furthermore, this method of assessing PT elongation has been shown to have a good test–retest reproducibility by us (Table 1) and others (Reeves et al. 2003).

**Table 1:** Test–retest reproducibility of patellar tendon measurements in eight healthy, recreationally active young men

Tendon property	Typical error (95% CI)	CV (%)	ICC (95% CI)
CSA (25% TL)	1.460 (0.966–2.972) mm^2^	1.234	0.997 (0.987–0.999)
CSA (50% TL)	1.685 (1.114–3.429) mm^2^	1.694	0.978 (0.898–0.995)
CSA (75% TL)	2.591 (1.713–5.273) mm^2^	1.996	0.988 (0.941–0.998)
Mean CSA	1.190 (0.787–2.421) mm^2^	1.004	0.996 (0.980–0.999)
Stress	0.4065 (0.269–0.827) MPa	0.968	0.996 (0.982–0.999)
Elongation	0.322 (0.213–0.657) mm	5.653	0.986 (0.935–0.997)
Strain	0.704 (0.465–1.432) %	5.506	0.986 (0.936–0.997)
Stiffness	63.6 (39.7–156.1) N/mm	4.477	0.988 (0.912–0.998)
Young’s modulus	0.032 (0.021–0.071) GPa	5.930	0.979 (0.889–0.996)

Measurements were taken on two separate occasions (at the same time of day) within a 12-day period. CSA measurements were taken at rest with the knee flexed at 90° (0° = full extension). Tendon stress, elongation, strain, stiffness, and Young’s modulus were all calculated using the highest common force (interval)

*CV* coefficient of variation, *ICC* intra-class correlation coefficient, *CSA* cross-sectional area, *TL* tendon length, *MPa* megapascals, *GPa* gigapascals

*P* < 0.001 for all ICCs

#### Antagonist muscle co-activation

Antagonist (hamstring) muscle co-activation during RMVC was assessed via electromyography (EMG) activity. The biceps femoris long head (BFlh) muscle [representative of the knee flexor muscle group (Kellis and Baltzopoulos 1997)] was identified via palpation during a submaximal knee flexion with the participant in the prone position. After preparing the skin surface (shaving, lightly abrading, and cleansing with 70% ethanol) at 2/3 muscle length from the proximal end [SENIAM guidelines (Freriks et al. 1999)], two bipolar Ag–AgCl surface electrodes (Neuroline, Medoicotest, Rugmarken, Denmark) were placed 20 mm apart along the sagittal axis of the muscle belly and a reference electrode placed on the lateral tibial condyle. The root mean square (RMS) of the BFlh EMG signal was recorded at each 10% knee extension R MVC and for 500 ms around the peak torque during a knee flexion MVC at 90° knee flexion. Assuming a linear relationship between EMG and torque output (Kellis and Baltzopoulos 1997), the torque generated by the antagonists during each 10% knee extension RMVC was estimated by dividing the BFlh EMG during knee-extensor RMVC by the BFlh maximal EMG (BFlh EMG during knee flexion MVC) and multiplying this ratio by the knee flexion MVC torque at each 10% RMVC. This torque was added to the knee-extensor torque value at the relevant 10% RMVC to provide the gross knee-extensor RMVC torque. Dividing the RMVC torque by the PT moment arm at 90° knee flexion [assumed to be 0.048 m based on MRI measurements in young healthy men (Erskine et al. 2009)] provided the PT force at each 10% RMVC. Due to technical issues with some EMG recordings, EMG data were only available in a subsample of elite (*n* = 12) and non-elite (*n* = 8) participants. As a limited sample size will negatively impact the statistical power of correlation analyses (Guadagnoli and Velicer 1988), and investigating the relationship between tendon properties and power during CMJs was one of the main aims of this study, we have reported tendon properties without accounting for antagonist muscle co-activation [which is not uncommon practice (Stenroth et al. 2012, 2015; Peltonen et al. 2012)] in an effort to maximise statistical power. However, to support these findings, we have also reported the results from correlation analyses in the subsample, where antagonist muscle co-activation was accounted for.

#### Analysis of tendon data

PT elongation was recorded as the distance the patella apex moved from the external marker every 10% RMVC. The video frame that corresponded to each 10% RMVC was exported as a portable network graphics (.png) file and the distance from the patella apex to the external marker was measured using image analysis software (ImageJ v. 1.47, National Institute of Health, MD, USA). Individual force–elongation curves were fitted with a second-order polynomial (*R*^2^ > 0.95 in all cases). Tendon stiffness (∆Force/∆PT length) values were obtained over the highest 20% common force interval in the weakest participant with the lowest maximal RMVC peak force (2599–3248 N). This approach, as performed by others (e.g., Seynnes et al. 2009), was necessary to avoid having to extrapolate some data points beyond the visible part of the force–elongation curve relationship (which would have been required for the weaker participants). It also allowed us to compare the mechanical and material properties of elite and non-elite player tendons under the same forces and conditions. However, we do acknowledge that this technique is limited, as we are assuming that the force elongation relationship between 2599–3248 N is linear (Pearson and Onambélé 2012). PT elongation was calculated as the change in tendon length at (3248 N) from resting length. Tendon strain was calculated as the change in tendon length at the highest common RMVC force level relative to the original tendon length (∆*L*/*L*_o_), and expressed as a percentage. Young’s modulus (*E*) was calculated by multiplying stiffness (*k*) with the ratio of the resting tendon length (*l*_0_) to mean tendon CSA, i.e., *E* = *k* × (*l*_0_/*s*).

### Test–retest reproducibility

To establish the test–retest reproducibility of our methods for measuring the morphological, mechanical, and material properties of the PT, we recruited an additional eight recreationally active, young, healthy men (age 28.0 ± 3.0 years; body mass 77.1 ± 7.4 kg; height 1.79 ± 0.04 m). All participants attended the laboratory on two separate occasions, at the same time of day, in the same standardised conditions as specified for the elite and non-elite soccer players, with a period of 7–12 days in between each testing session. Measurements were performed by the same researcher, as described in the “Tendon morphology”, “Tendon elongation during RMVC”, and “Analysis of tendon data” sections. Inter-day reliability for each measurement was expressed as typical error (TE), coefficient of variation (CV), and intra-class correlation coefficient (ICC, model: two-way mixed; type: absolute agreement) with 95% confidence intervals (CIs). These data are presented in Table 1, and for all variables, the TE and CV were low, and the ICC was high (≥ 0.979) with narrow CIs, thus indicating that our methods were highly reproducible.

### Statistical analyses

The mean and standard deviation (*s*) were calculated for all variables in both the whole cohort, and a subsample where antagonist co-activation was accounted for. All data were tested for normality using the Shapiro–Wilks normality test. For tendon CSA, tendon sCSA, and tendon elongation at each 10% RMVC interval, a two-way mixed ANOVA was used to determine a main effect of athlete status (between factor: elite *vs*. non-elite soccer players), tendon location [within factor: CSA at 0, 25, 50, 75, and 100% tendon length (this includes mean tendon CSA comparison)] or tendon elongation (within factor: tendon elongation at 10, 20, 30, 40, 50, 60, 70, 80, 90, and 100% RMVC), and an athlete status × tendon location, or athlete status × tendon elongation at a specific % of RMVC, interaction. If a significant interaction occurred, simple main effects and pairwise comparisons with Bonferroni adjustment were performed to reveal differences. If a significant main effect existed for tendon CSA location or tendon elongation at a specific % of RMVC, Bonferroni post hoc tests were used to establish the difference in CSA or tendon elongation between tendon locations or % RMVC force levels, respectively. For tendon force, length, stiffness, elongation, strain, and Young’s modulus, independent *t* tests were used to determine differences between elite and non-elite soccer players, in both the whole cohort and subsample (where antagonist co-activation was measured). Pearson’s correlations were used to determine relationships between jump performance variables (height or projectile range, peak V-power, peak H-power, or peak M-power) and tendon mean CSA and CSA at 0, 25, 50, 75, and 100% tendon length, strain, stiffness, and Young’s modulus. Statistical analysis was completed using SPSS version 21 (SPSS Inc., Chicago, IL, USA) and the significance level was set at *P* ≤ 0.05.

## Results

### Anthropometry

Elite soccer players were significantly taller (*P* = 0.009) and had significantly longer femur length (46.8 ± 2.1 vs. 44.0 ± 1.7 cm; *P* = 0.001) than non-elite. However, there was no difference in body mass between elite and non-elite soccer players (*P* = 0.267).

### Differences in patellar tendon properties between elite and non-elite players

#### Tendon morphology

**Fig. 1:**
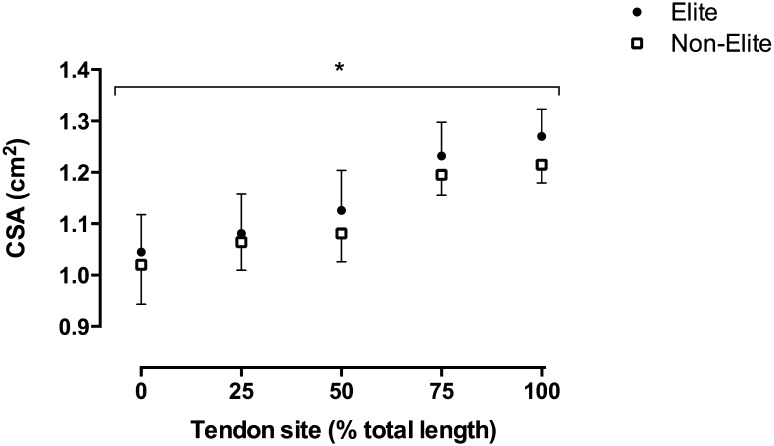
Patellar tendon cross-sectional area (CSA) along its length at 25% intervals in elite (*n* = 16, circles, mean + SD) and non-elite (*n* = 13, open squares, mean − SD) players. *Main effect, elite players significantly greater CSA than non-elite players (*P* < 0.05)

For tendon CSA, there was a main effect of athlete status, with elite soccer players demonstrating ~ 2.6% greater mean CSA (*F*_1,27_ = 4.439, *P* = 0.045; Fig. 1; Table 2). There was also a main effect of tendon location (*F*_4,108_ = 105.36, *P* < 0.001) with pairwise post hoc analyses, revealing that tendon CSA varied along the length of the tendon (0 vs. 25%, *P* = 0.001; 0 vs. 50%, *P* < 0.001; 0 vs. 75%, *P* < 0.001; 0 vs. 100%, *P* < 0.001; 25 vs. 75%, *P* < 0.001; 25 vs. 100%, *P* < 0.001; 50 vs. 75%, *P* < 0.001; 50 vs. 100%, *P* < 0.001; 75 vs. 100%, *P* = 0.007; Fig. 1); except at 25 vs. 50% tendon length where there was a non-significant difference (*P* = 0.063; Fig. 1). There was no significant interaction between tendon CSA and athlete status (*F*_4,96_ = 0.720, *P* = 0.565). Tendon resting length did not differ between groups (*t* = 0.119; *P* = 0.906; Table 2).

**Table 2:** Patellar tendon morphological, mechanical, and material properties calculated in the whole cohort [elite (*n* = 16) and non-elite (*n* = 13)]; mean ± SD

Tendon property	Elite	Non-elite
Mean CSA (mm^2^)	115 ± 6*	112 ± 3
Mean sCSA (mm^2^/kg^0.67^)	6.35 ± 0.44	6.41 ± 0.02
Resting tendon length (mm)	58.5 ± 7.1	58.2 ± 4.4
Stiffness (N/mm)	1269 ± 607	1707 ± 792
Elongation (mm)	6.83 ± 1.87*	4.92 ± 1.88
Strain (%)	11.73 ± 3.25**	8.38 ± 3.06
Stress (MPa)	28.29 ± 1.34*	29.15 ± 0.72
Young’s modulus (GPa)	0.64 ± 0.31	0.88 ± 0.39
MVC tendon force (N)	5728 ± 1522	4800 ± 989

*CSA* cross-sectional area, *sCSA* cross-sectional area isometrically scaled to body mass^0.67^, *MVC* maximal voluntary contraction

*A significant difference when comparing elite and non-elite (*P* < 0.05)

**A significant difference between elite and non-elite (*P* < 0.01)

For tendon sCSA, there was a main effect of tendon location (*F*_4,108_ = 106.07, *P* < 0.001) with pairwise post hoc analyses, revealing that tendon CSA varied along the length of the tendon (0 vs. 25%, *P* = 0.024; 0 vs. 50%, *P* < 0.001; 0 vs. 75%, *P* < 0.001; 0 vs. 100%, *P* < 0.001; 25 vs. 75%, *P* < 0.001; 25 vs. 100%, *P* < 0.001; 50 vs. 75%, *P* < 0.001; 50 vs. 100%, *P* < 0.001; 75 vs. 100%, *P* = 0.031); except at 25 vs. 50% tendon length where there was a non-significant difference (*P* = 0.185). However, there was no significant main effect of athlete status (elite 0.063 ± 0.004 mm/kg^0.67^; non-elite 0.063 ± 0.004 mm/kg^0.67^, *F*_1,27_ = 0.006, *P* = 0.941) and no significant interaction between athlete status and tendon CSA location (*F*_4,108_ = 0.720, *P* = 0.580). Furthermore, tendon resting length relative to femur length did not differ between groups (*t* = − 1.520; *P* = 0.140).

#### Tendon mechanical and material properties

**Fig. 2:**
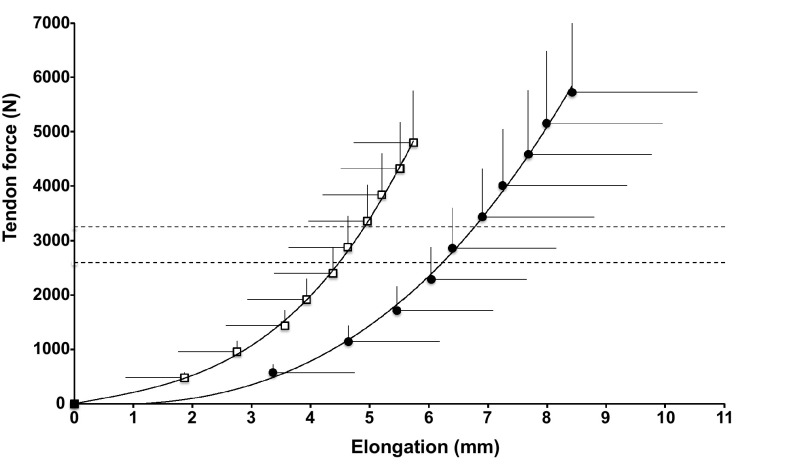
In vivo patellar tendon force–elongation relation in elite (*n* = 16; circles) and non-elite (*n* = 13; open squares) soccer players. Significant main effect for athlete status (*P* < 0.05). Data are mean + SD

We present differences in the tendon mechanical and material properties between elite and non-elite soccer players calculated in both the whole cohort (elite: *n* = 16; non-elite: *n* = 13) and subsample (elite: *n* = 12; non-elite, *n* = 8) where antagonist co-activation was accounted for. Elite soccer players demonstrated significantly greater tendon elongation and strain than non-elite players (whole cohort: elongation, *P* = 0.011; strain, *P* = 0.009; Table 2; subsample: elongation, 6.72 ± 1.94 vs. 4.76 ± 1.97 mm, *P* = 0.041; strain, 11.38 ± 3.27 vs. 8.03 ± 3.25%, *P* = 0.037). Moreover, when we compared tendon elongation at each 10% RMVC interval in the whole cohort, there was a significant main effect of athlete status (*F*_9,243_ = 93.00, *P* < 0.001, Fig. 2) and tendon elongation (*F*_9,27_ = 11.13, *P* < 0.001, Fig. 2), but no significant interaction effect between tendon elongation and athlete status (*F*_9,243_ = 1.63, *P* = 0.108). This finding suggests that elite soccer player tendon elongation was greater than non-elite soccer players at all 10% levels of RMVC. However, there were no differences between elite and non-elite soccer players regarding tendon stiffness (whole cohort: *P* = 0.104; Table 2; subsample: *P* = 0.496). There was a non-significant tendency for Young’s modulus (*P* = 0.075; Table 2) to be lower and maximum tendon force (*P* = 0.069; Table 2) to be higher in elite compared to non-elite soccer players in the whole cohort. In the subsample, elite soccer players produced significantly greater maximum tendon force in comparison to non-elites (6374 ± 1658 vs. 4817 ± 607 N, *P* = 0.021).

For a comparison of unilateral jump performance between elite and non-elite soccer players, please refer to our data presented in a previously published study specifically aimed to determine whether jump performance could distinguish between soccer playing levels (Murtagh et al. 2017).

### Relationships between patellar tendon properties and direction-specific jump performance

The positive and inverse relationships between jump performance variables and tendon properties from the whole cohort are displayed in Table 3. Additional figures have been included to illustrate the spread of data for specific relationships. In the subsample, correlations between jump performance variables and tendon properties are also illustrated in the text.

**Table 3:**
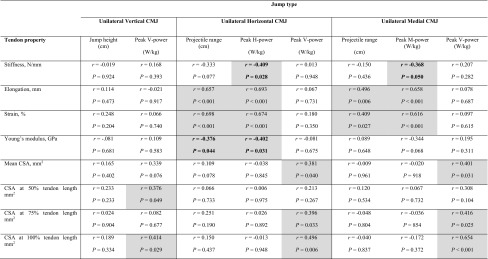
Correlations between unilateral countermovement jump (CMJ) performance measures and patellar tendon properties in elite (*n* = 16) and non-elite (*n* = 13) soccer players

Significant correlations are highlighted in grey. Significant inverse correlations are highlighted in bold

*CSA* cross-sectional area, *peak V-power* peak vertical power isometrically scaled to body mass^0.67^, *peak H-power* peak horizontal power isometrically scaled to body mass^0.67^, *peak M-power* peak medial power isometrically scaled to body mass^0.67^

**Fig. 3:**
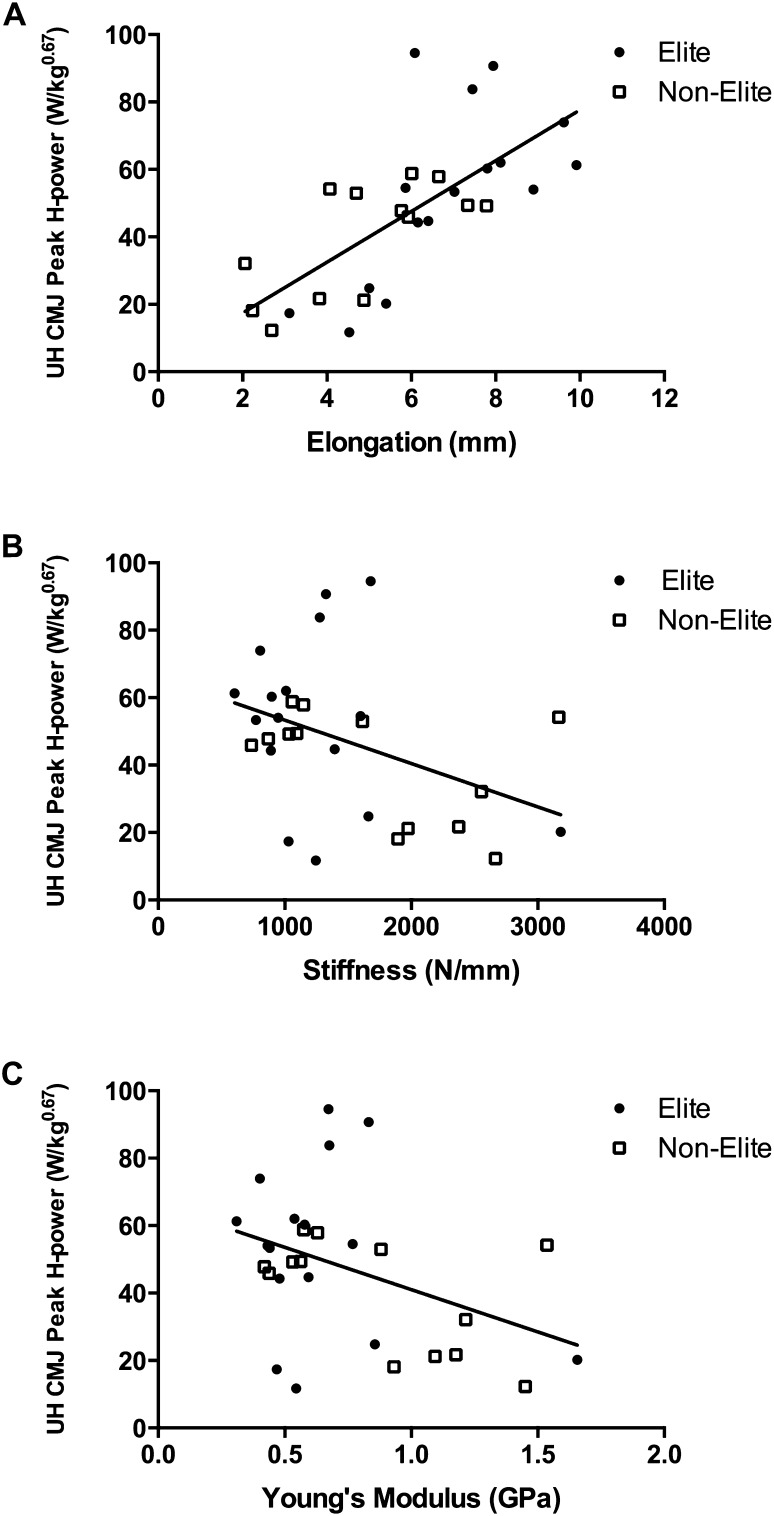
Relationship between unilateral horizontal (UH) countermovement jump (CMJ) peak H-power and tendon elongation (**a**, *r* = 0.693, *P* < 0.001), stiffness (**b**, *r* = − 0.409, *P* = 0.028), and Young’s Modulus (**c**, *r* = − 0.402, *P* = 0.031) in elite (*n* = 16, circles) and non-elite (*n* = 13, open squares) players. *Peak H-power* peak horizontal power isometrically scaled to body mass^0.67^

**Fig. 4:**
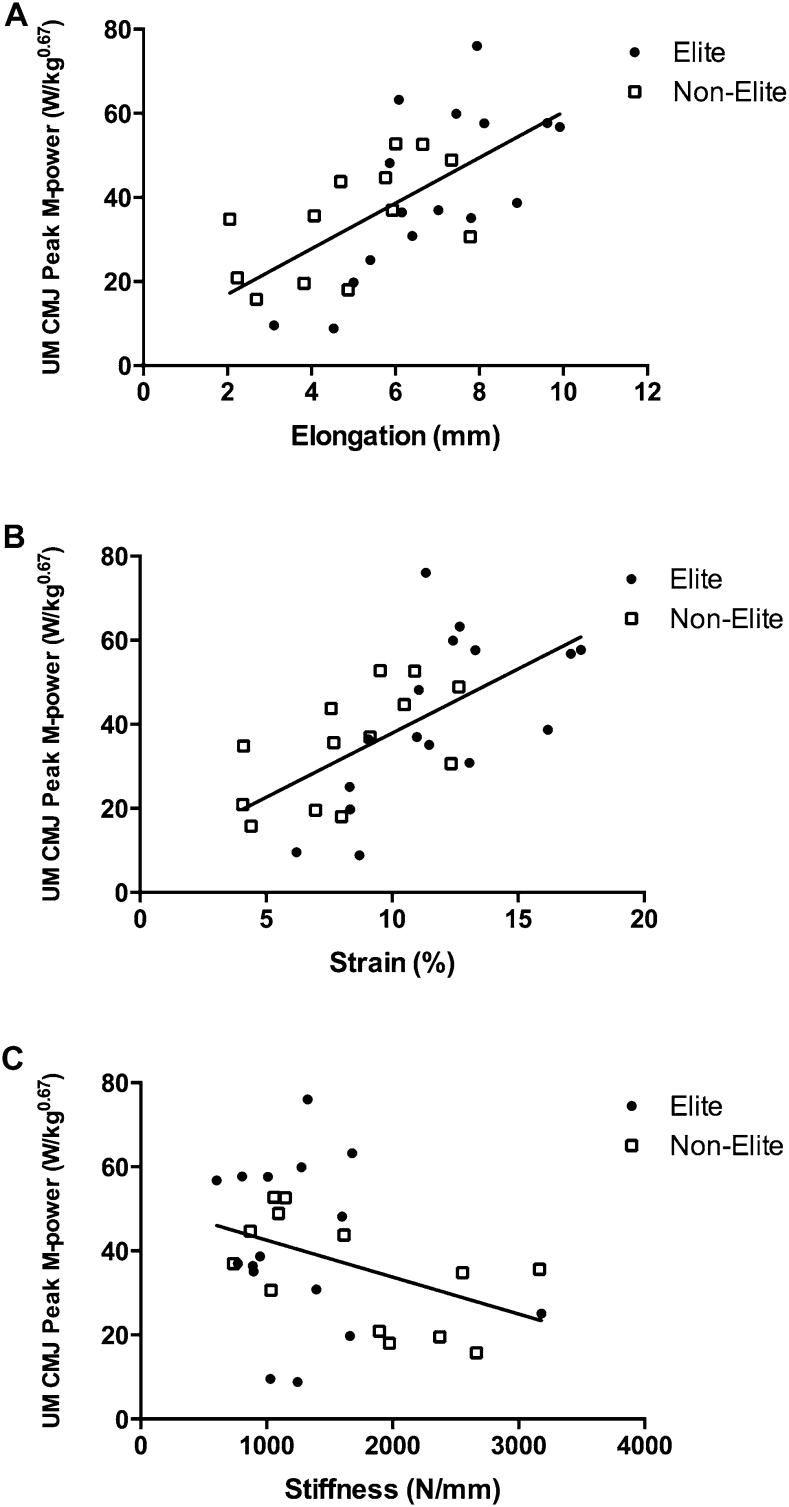
Relationship between unilateral medial (UM) countermovement jump (CMJ) peak M-power and tendon elongation (**a**, *r* = 0.658, *P* < 0.001), strain (**b**, *r* = 0.616, *P* < 0.001), and stiffness (**c**, *r* = − 0.368, *P* = 0.050) in elite (*n* = 16, circles) and non-elite (*n* = 13, open squares) players. *Peak M-power* peak medial power isometrically scaled to body mass^0.67^

In the whole cohort and subsample, unilateral horizontal-forward jump performance variables correlated positively with tendon elongation (whole cohort: Fig. 3a; Table 3; subsample: projectile range, *r* = 0.733, *P* < 0.001, peak H-power: *r* = 0.734, *P* < 0.001) and strain (whole cohort: Table 3; subsample: projectile range: *r* = 0.765, *P* < 0.001, peak H-power: *r* = 0.677, *P* = 0.001), but inversely with tendon stiffness (whole cohort: Fig. 3b; Table 3; subsample: projectile range: *r* = − 0.576, *P* = 0.008, peak H-power: *r* = − 0.579, *P* = 0.007) and Young’s modulus (whole cohort: Fig. 3c; Table 3; subsample: projectile range: *r* = − 0.559, *P* = 0.010, peak H-power: *r* = − 0.490, *P* = 0.028). Similarly, unilateral medial CMJ performance variables correlated positively with tendon elongation (whole cohort: Fig. 4a; Table 3; subsample: projectile range: *r* = 0.513, *P* = 0.021, peak M-power: *r* = 0.633, *P* = 0.003) and strain (whole cohort: Fig. 4b; Table 3; subsample: peak M-power: *r* = 0.570, *P* = 0.009), but inversely with tendon stiffness (whole cohort: Fig. 4c; Table 3; subsample: tendency for a significant correlation with peak M-power: *r* = − 0.432, *P* = 0.057). Mean tendon CSA, CSA at 75% and CSA at 100% correlated positively with unilateral horizontal-forward CMJ peak V-power and unilateral medial CMJ peak V-power (Table 3) in the whole cohort. Furthermore, tendon CSA at 50 and 100% correlated positive with unilateral vertical jump peak V-power (Table 3). There were no significant correlations between any jump variable and tendon CSA at 0 or 25% (*r* ≤ 0.307; *P* ≥ 0.116) (Table 3).

## Discussion

The aims of our study were to compare PT properties between elite and non-elite soccer players, and to establish if these properties were related to unilateral jump performance in different directions. Our results showed that tendon CSA, elongation and strain were greater in elite soccer players. These specific tendon properties may, therefore, be considered determinants of elite soccer playing status. Tendon elongation and strain also correlated positively with peak horizontal power and medial power during unilateral horizontal and unilateral medial CMJ, respectively, while tendon stiffness and Young’s modulus correlated inversely with horizontal power during unilateral horizontal CMJ. Only PT CSA at 50% and 100% tendon length was related to unilateral vertical CMJ performance. Our study suggests that a more compliant PT facilitates greater unilateral CMJ performance in the horizontal-forward and medial directions, but PT stiffness does not contribute to vertical CMJ performance.

Comparing the physiological capabilities in soccer players competing at different performance levels may provide an insight into the physiological factors that determine elite soccer playing status. Tendon CSA was significantly greater in elite compared to non-elite soccer players at all tendon locations (main effect of athlete status and no significant interaction between athlete status and tendon CSA). Although the contents of elite and non-elite player habitual weekly training schedules seem similar (please refer to the “Methods” section), elite soccer players perform a higher volume of soccer training sessions and, unlike non-elite players, also partake in non-resistance-based gym training. Elite players are, therefore, exposed to a greater number of cyclic lower limb loading cycles and high impact explosive actions per week, both of which have been shown to potentially stimulate increments in tendon CSA (Magnusson and Kjaer 2003; Couppe et al. 2008; Wiesinger et al. 2016). Subsequently, our results may agree with the previous literature documenting the relationship between habitual daily loading and tendon size (see Wiesinger et al. 2015 for review), thus potentially suggesting that long-term exposure to elite soccer training results in greater PT tendon CSA. However, when tendon CSA was normalised to body mass^0.67^, there was no significant difference in tendon CSA between cohorts at any tendon location (no significant main effect of athlete status and no significant interaction between athlete status and tendon CSA). Although we found no significant difference in body mass^0.67^ between the elite and non-elite players, this approach was necessary due to the close relationship found previously between body mass and tendon CSA (Rosager et al. 2002). Elite players were significantly taller than non-elites and the difference in tendon CSA between elite and non-elite players may, therefore, be related to individual differences in body size rather than adaptations to chronic habitual loading patterns. Interestingly, in agreement with other literature (Wang et al. 2013), although elite players displayed greater stature and femur length compared to non-elites, there was no difference in resting tendon length, thus potentially suggesting that the length of the PT in soccer players is not related to their body dimensions. Nevertheless, without a control group (who did not partake in soccer-specific training) to compare with, or longitudinal training study, it still remains unknown if long-term soccer training stimulates PT CSA adaptation.

Despite greater tendon CSA, tendon strain was higher in elite compared to non-elite soccer players. This illustrates that elite soccer players have PT structures that elongate more, relative to their original length, compared to non-elites at a given tendon force value (highest common tendon force = 3248 N). Although elite soccer players displayed greater PT elongation, we found no significant difference in tendon stiffness between elite and non-elite soccer players. This suggests that when specific forces (representative of the common highest 20% force interval of the weakest participant: 2599–3248 N) were applied to the PT, there was no significant difference in the change in elongation between elite and non-elite players. It, therefore, appears that the greater magnitude of difference in PT elongation between elite and non-elite players occurs at lower force levels (below the common force interval of the weakest participant). Young’s modulus (i.e., the relation between stress and strain), which represents the material properties of the tendon independent of its dimensions, provides a more accurate representation of the in vivo function of the PT when comparing elite and non-elite soccer players (Foster et al. 2014). Our findings showed that there was a tendency for elite soccer players to display lower Young’s modulus than non-elite players. The greater elongation and strain demonstrated by elite in comparison to non-elite soccer players may, therefore, be due to differences in the microstructure of the tendon, including less collagen cross linking and less fibril packing (Reed and Iozzo 2002). Moreover, as the major difference in the change in PT elongation between elite and non-elite players occurs at lower force levels, it may be that elite soccer players display PTs with more collagen crimping (Kastelic et al. 1980). However, given the non-significant difference in Young’s modulus between groups, this theory remains speculative.

Considering the sprint demands of elite soccer match-play (Bradley et al. 2010), the greater PT elongation in elite compared to non-elite soccer players in our study is consistent with previous studies, reporting that the VL tendon–aponeurosis complex of elite sprinters elongates more than both non-elite sprinters (Stafilidis and Arampatzis 2007) and untrained participants (Kubo et al. 2011). While it has been well documented that resistance and plyometric training interventions increase PT stiffness (see Wiesinger et al. 2015 for review), no specific training intervention has been shown to increase the elongation properties of the PT structures (Seynnes et al. 2009; Reeves et al. 2003), and only bed rest (Reeves et al. 2005), detraining (Kubo et al. 2010), and ageing (Reeves et al. 2003) are known to induce these changes. Therefore, as the CC genotype of the COL5A1 (rs12722) genetic variant (which encodes the pro alpha chain of the type V collagen) has previously associated with greater PT elongation and lower stiffness (Kubo et al. 2013), it is possible that elite soccer players have a genetic predisposition to tendon structures with greater elongation properties compared to non-elite soccer players. However, the research associating specific genetic variants and PT properties remains inconclusive (Foster et al. 2014) and it cannot be discounted that certain types or combinations of long-term soccer development training stimuli, which have not been investigated to date, could induce such tendon adaptations. Nevertheless, our findings highlight the potential importance of assessing PT elongation in elite soccer talent selection and development protocols.

Unilateral CMJ tasks oriented in different directions have been suggested to represent a measurement of soccer-associated power (Murtagh et al. 2017), and the relationship between tendon properties and unilateral CMJ performance in different directions are, therefore, of interest for the assessment and development of muscular power in elite soccer players. Our study showed that in soccer players, PT elongation and strain were both positively related to unilateral horizontal-forward CMJ peak H-power and unilateral medial CMJ peak M-power, while tendon stiffness was inversely related to unilateral horizontal-forward CMJ peak H-power and unilateral medial CMJ peak M-power. These findings suggest that more compliant PTs facilitate greater unilateral horizontal and unilateral medial CMJ performance in soccer players. Moreover, Young’s modulus was inversely related with unilateral horizontal CMJ projectile range and peak H-power, thus suggesting that a tendon with more elastic properties enhances unilateral horizontal CMJ performance.

As acceleration and sprinting activities require the production of high levels of horizontal-forward power (Buchheit et al. 2014), these findings are in accordance with the previous studies reporting that 100 m sprint performance is positively related to VL aponeurosis compliance (Kubo et al. 2000b) and maximal elongation (Stafilidis and Arampatzis 2007). Real-time ultrasonography observations of tendon behaviour in vivo has shown that, during high-intensity jumping movements whereby the range of joint motion is small, the VL muscle fascicles lengthen only marginally, if at all during the eccentric phase (Finni et al. 2003), and are thought to function quasi-isometrically. Horizontal-forward CMJs have been shown to require ~ 10° less knee flexion than vertical CMJs (Fukashiro et al. 2005). Hence, unilateral horizontal-forward and possibly unilateral medial CMJs may require a quasi-isometric contraction of the knee-extensor muscle group, inducing greater tendon lengthening (Reeves and Narici 2003), thus allowing the tendon to store more potential energy and recoil at greater speeds. This would enable the tendon to act as a power amplifier during horizontal-forward and medial CMJs (Nagano et al. 2004). Moreover, it has also been reported that during the initial concentric phase of stretch-shortening cycle exercises, the rapid shortening of the tendon contributes to lowering the shortening velocity of the muscle fibres to near isometric conditions (Kawakami et al. 2002). Therefore, a more compliant PT will have a capacity for greater elongation, allowing the knee-extensor muscle fibres more time to develop greater forces during the concentric propulsive phase of sprinting (Kubo et al. 2011), unilateral horizontal-forward CMJ and unilateral medial CMJ activities. Hence, our data support a notion that a more compliant PT could enhance soccer player performance levels during activities that depend heavily upon horizontal push-off capacity.

While tendon CSA was not related to unilateral horizontal-forward or medial CMJ peak H-power or M-power, respectively, distal tendon CSA was positively associated with the ability to produce peak vertical power during unilateral vertical, horizontal and medial CMJs (Table 3). However, there was no relationship between any mechanical or material PT properties with peak V-power during any CMJ. We have previously shown that unilateral vertical and medial CMJ peak V-power is positively related to quadriceps femoris muscle size (Murtagh et al. 2018). Considering the relationship between body size and tendon CSA (Rosager et al. 2002), players with larger quadriceps may also have greater PT CSA. Hence, the ability to produce peak V-power may be more closely related to the neuromuscular properties of the knee extensors rather than the properties of the PT and this may be the reason why we found a positive relationship between tendon CSA and peak V-power. The lack of direct relationships between PT mechanical and material properties and unilateral vertical CMJ performance in the soccer players in our study is in accordance with some (Kubo et al. 2000a), but not all the previous studies (Bojsen-Møller et al. 2005). Bojsen-Møller et al. (2005) found that a stiffer VL tendon–aponeurosis complex contributed to enhanced peak force and power outputs during high-intensity isometric and dynamic bilateral vertical jumping tasks, respectively. Discrepancies between the findings of Bojsen-Møller et al. (2005) and our results are likely due to methodological disparities. Whilst we analysed ultrasound images of the PT, Bojsen-Møller et al. (2005) measured the VL aponeurosis and, therefore, approximated tendon elongation. Consequently, it is possible that their stiffness measurements do not account for total displacement of the tendon and may underestimate tendon compliance.

We do acknowledge some limitations to our study that could inform future research. Our investigation only includes elite players from one soccer club and should be replicated in a wider range of soccer institutions. Moreover, we have not directly investigated the capacity of the tendon to store and release energy in vivo and future research should also measure tendon hysteresis [as performed by Wiesinger et al. (2017)]. The loading rates during the ramped isometric voluntary contractions were dependent on the participants maximal voluntary force level. The previous literature shows that there are differences in the stiffness and Young’s modulus calculations when ramped isometric contractions are performed at 50 N m/s compared to 80 and 110 N m/s (Kösters et al. 2014). Subsequently, we cannot discount that the different loading rates may have affected calculations of stiffness and Young’s modulus. However, as the loading rates between participants in our study (29.7–71.3 N m/s) are within a narrow range, we believe it is unlikely that this method of assessment compromised the validity of our data. Moreover, as the previous studies adopted similar methodologies (Hansen et al. 2006; Seynnes et al. 2009; Malliaras et al. 2013), we believe that our data are comparable to current research studies.

Our study suggests that novel soccer talent selection protocols should include a measurement of PT properties, and elite soccer clubs should aim to recruit, and develop, players with more elastic/compliant PTs. As elite players displayed greater absolute tendon CSA, our research suggests that long-term elite soccer training stimulates such adaptations and may support the hypothesis of functionally driven tendon adaptation proposed by Wiesinger et al. (2016). However, future research is needed to confirm this theory as when we accounted for body size, no significant difference between elite and non-elite players was found. We also show that a more compliant PT appears to facilitate horizontal-forward and medial jump performance, and therefore, practitioners aiming to develop unilateral explosive performance in these directions should, perhaps, reconsider the prescription of training interventions that may induce a stiffening of the PT. We recommend that future research should also investigate training interventions that may increase PT elasticity.

## Conclusion

We have shown that patellar tendon CSA, elongation, and strain are greater in elite compared to non-elite soccer players, and are, therefore, important indicators of U18 and U21 elite soccer playing status. More compliant patellar tendon properties were positively related to performance in jumps with a horizontal push-off, yet they were not related to vertical jump performance. We show that a more compliant patellar tendon will likely benefit elite soccer players across all soccer-specific activities involving a powerful single-leg push off in the horizontal direction.

## Abbreviations

BFlh: Biceps femoris long head
CMJ: Countermovement jump
CSA: Cross-sectional area
sCSA: Cross-sectional area normalised to body mass^2/3^
EMG: Electromyography
PT: Patellar tendon
PCSA: Physiological cross-sectional area
Peak H-power: Peak horizontal power normalised to body mass^2/3^
Peak M-power: Peak medial power normalised to body mass^2/3^
Peak V-power: Peak vertical power normalised to body mass^2/3^
RMVC: Ramped maximal voluntary contraction
VL: Vastus lateralis
